# Shedding Light on Hidradenitis Suppurativa Activity: A Pilot Study to Evaluate the Potential of [^99m^Tc]Tc-Anti-TNF-Alpha Scintigraphy

**DOI:** 10.3390/ph18081190

**Published:** 2025-08-12

**Authors:** José Marcos Telles da Cunha, Gabriel Gutfilen-Schlesinger, Beatriz Moritz Trope, Flávia Paiva Proença Lobo Lopes, Sergio Augusto Lopes de Souza, Bianca Gutfilen

**Affiliations:** 1 Department of Internal Medicine, Dermatology Service, Medical School, Universidade Federal do Rio de Janeiro, Rio de Janeiro 21941-913, Brazil; jmtcunha@yahoo.com.br; 2 Laboratório de Marcação de Células e Moléculas (LMCM), Radiology Department, Medical School, Universidade Federal do Rio de Janeiro, Rio de Janeiro 21941-913, Brazil; gabriel.gut.sch@gmail.com (G.G.-S.); flaviapaivalopes@medicina.ufrj.br (F.P.P.L.L.); sergioalsouza@medicina.ufrj.br (S.A.L.d.S.); 3 Dermatology Service, Hospital Universitário Clementino Fraga Filho, Universidade Federal do Rio de Janeiro, Rio de Janeiro 21941-913, Brazil; biatrope@gmail.com

**Keywords:** hidradenitis suppurativa, scintigraphy, anti-TNF-alpha, diagnosis

## Abstract

Shedding light on Hidradenitis Suppurativa activity: a pilot study to evaluate the potential of [^99m^Tc]Tc-anti-TNF-alpha scintigraphy. **Background/Objectives**: Hidradenitis suppurativa (HS), also known as acne inversa and Verneuil’s disease, is a chronic and non-contagious auto-inflammatory disease of pilo-sebaceous units that can lead to severe complications and sequelae. The actual prevalence of HS is unknown due to diagnostic delay and/or misdiagnosis, but it is estimated to affect 0.00033–4.1% of the general population worldwide. Only severe cases are referred for imaging assessment, and the final diagnosis is mostly established on a clinical basis. Here, we present a pilot study aiming to evaluate clinically active inflammatory disease in patients with HS using [^99m^Tc]Tc-anti-TNF-alpha scintigraphy. **Methods**: Four patients (2 male, 2 female) had their HS clinical features measured through the HS-PGA and Hurley Score and compared to [^99m^Tc]Tc-anti-TNF-alpha scintigraphy findings. **Results**: Scintigraphy with [^99m^Tc]Tc-anti-TNF-alpha showed abnormal uptake in clinically active lesions of HS and also detected some clinically unknown potential active lesions, later confirmed by clinical reassessment after the imaging. **Conclusions**: Our results suggest that scintigraphy with [^99m^Tc]Tc-anti-TNF-alpha may be able to detect increased radiotracer uptake in most clinically identified active lesions in this pilot cohort. While promising, due to the inherent limitations of this pilot study, more studies need to be carried out to arrive at a definitive diagnostic assessment of active inflammatory disease using this method.

## 1. Introduction

Hidradenitis Suppurativa (HS; global estimates vary from 0.00033 to 4.1%) [1] belongs to a group of hair follicle occlusion syndromes, which also includes acne conglobata, dissecting cellulitis, and pilonidal sinus. Since they share the same pathophysiological process, subjects can present these conditions simultaneously [2].

The pathogenesis of this condition is primarily attributed to the entrapment of keratin within the hair follicle, which is presumed to be incompletely degraded, likely due to dysfunctional gamma-secretase enzyme activity. This leads to follicular rupture and subsequent spillage of its contents into adjacent tissues. This cascade elicits a robust pro-inflammatory response, characterized by the infiltration of macrophages, neutrophils, and lymphocytes, and the concerted action of metalloproteinases, chemokines (including CXCL-8, CCL-4, CXCL-10, and CCL-26), and cytokines (such as IL-1, IL-17, IL-20, IL-22, IL-23, and TNF-alpha) [1,3,4,5,6]. While non-specific in isolation, these cytokines collectively contribute substantially to the inflammatory cascade. The potential for these molecules to serve as biomarkers is therefore of considerable interest. TNF-alpha, a cytokine predominantly synthesized by activated macrophages, is involved in a spectrum of biological activities, including the induction of pro-inflammatory events, stimulation of fibroblast growth, mediation of cytotoxic or cytostatic effects, activation of endothelial cells, and promotion of IL-8 synthesis [5,7] and is observed in the pathophysiology of HS.

Recently, immunobiological agents have been increasingly used to target these inflammatory molecules. In this manner, they have revolutionized the management of inflammatory diseases such as inflammatory bowel diseases, arthritis, and psoriasis. This has enabled a transition from typically broad-spectrum anti-inflammatory approaches to more specific, targeted therapeutic strategies [8]. Anti-TNF-alpha agents approved in severe HS are adalimumab, with proven efficacy, and infliximab, which allows for posology adjustment according to individual patient needs, an additional advantage in the current context of pursuing personalized medicine. Limited clinical data are available regarding the use of other anti-TNF-alpha agents for this indication, such as certolizumab pegol and golimumab [9]. Despite promising results with these agents, real-world and retrospective studies are investigating which patients are more likely to achieve a therapeutic response and the paradoxical results observed in some patients. Initial studies suggest a potential link to the inflammatory status at the time of treatment initiation, indicating that further research is required to understand the immunopathogenesis of the disease [9,10,11]. In this context, the potential use of radioactively labeled anti-TNF-alpha agents as radiopharmaceuticals in the assessment of HS patients may also aid in screening potential treatment responders.

The accurate diagnosis of active inflammatory lesions in patients with HS and who present severe inflammatory conditions is a significant challenge for clinicians. This underscores the urgent need for more sensitive and specific imaging methods. In the evolving era of theranostics, where the goal is to identify specific biomarkers for diagnosis and subsequently utilize the same biomarker for therapeutic purposes, the use of monoclonal antibodies, such as anti-TNF agents, offers a promising avenue. When radioactively labeled, these agents can serve as diagnostic tools capable of informing therapeutic decisions and guiding disease management [12]. Infliximab (a chimeric antibody) and adalimumab (a humanized antibody), both anti-TNF-alpha monoclonal antibodies, represent commonly employed agents in clinical practice. Adalimumab, a fully humanized anti-TNF-alpha agent, was initially approved for the treatment of psoriasis and rheumatoid arthritis. Subsequent studies have confirmed its efficacy in inflammatory bowel disease, ankylosing spondylitis and, more recently, HS [13].

Given the encouraging outcomes observed with [^99m^Tc]Tc-anti-TNF-alpha scintigraphy in assessing active inflammation in other diseases, such as rheumatoid arthritis [14,15], ankylosing spondylitis [16], Graves’ ophthalmopathy [17,18], and more recently in psoriatic arthritis [19], we aim to evaluate its application in HS. We now present a pilot study designed to detect clinically active inflammatory sites in patients with HS using [^99m^Tc]Tc-anti-TNF-alpha scintigraphy.

## 2. Results

Four patients fulfilling the inclusion criteria volunteered to participate. Baseline characteristics of the included patients are presented in Table 1.

### 2.1. Radiochemical Control

In adherence to institutional protocol, a Radiochemical Quality Control (RQC) test was performed for each patient before radiopharmaceutical injection, using previously described techniques [20,21]. This test used ascending instant thin-layer chromatography (ITLC) on Whatman No. 4 paper. The mobile phases for this RQC were 0.9% NaCl and acetone.

To perform the ITLC, a 10 µL sample was applied to the stationary phase, which consisted of a 1.0 cm by 10 cm paper strip.

Using 0.9% NaCl as the mobile phase, over 98% of the radiolabeled compound remained at the origin. When acetone was used as the mobile phase, over 98% of the radiolabeled compound also remained at the origin, demonstrating no free [^99m^Tc]pertechnetate.

Administration was permitted only when a labeling efficiency of 90% or higher was achieved. This test consistently demonstrated a labeling efficacy greater than 98% in all cases, confirming the absence of both free [^99m^Tc]pertechnetate and hydrolyzed-reduced [^99m^Tc].

### 2.2. Adverse Drug Reaction

[^99m^Tc]Tc-anti-TNF-alpha was well tolerated throughout the study group. No adverse drug reaction was reported by patients or clinically identified during the subsequent outpatient appointment. No clinical assays were performed, given the absence of recorded clinical alterations.

### 2.3. Demographic Data

Four patients with mean age 41 (±7.14) years were evaluated, with respective gender, clinical features and severity scores presented in Table 1.

### 2.4. Case Reports

Patient 1 (P1)

**Figure 1:**
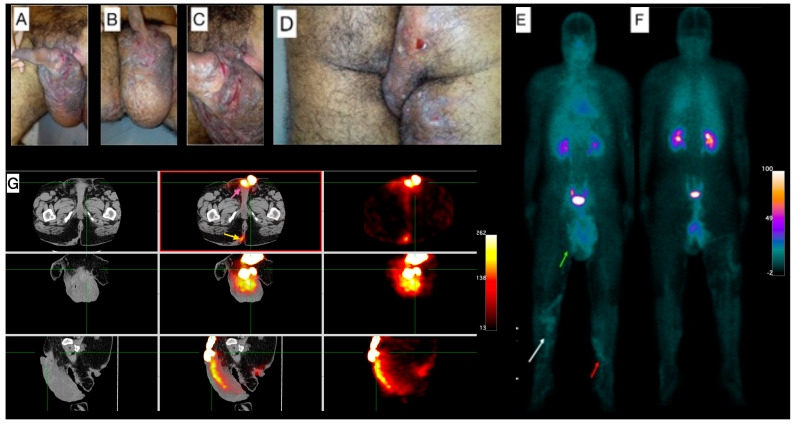
Patient 1 clinical and imaging findings. (**A**) External genitalia and inguinal region skin lesions. Noticeable lymphedema in the scrotum. (**B**) Lengthy fissures on the basis of the penis, and (**C**) lymphangiectasia on the scrotum’s skin. (**D**) Gluteal region shows pronounced edema, hyperchromia, fistulae, and lichenification, extending into the proximal third of the thighs. (**E**) Whole-body [^99m^Tc]Tc-anti-TNF-alpha scintigraphy, anterior view, demonstrates abnormal uptake in the scrotum (green arrow). A venous tract is visible in the distal thigh extending to the proximal third of the right inferior limb (white arrow) and the proximal third of the left lower limb (red arrow). (**F**) The posterior view of the scintigraphy further confirms abnormal uptake in the proximal third of the right thigh. (**G**) Tomographic images include a Computed Tomography (CT) scan on the left panel, fused Single-Photon Emission Computed Tomography/Computed Tomography (SPECT/CT) images in the center, and SPECT images on the right. These reveal abnormal uptake in the scrotum (pink arrow; top-center image) and the right intergluteal cleft (yellow arrow; bottom-center image).

Male, 38-year-old, diagnosis of HS for more than 20 years, using antibiotics (rotation scheme: cephalexin or sulfamethoxazole + trimethoprim or doxycycline) for approximately two years. Clinical exam showed lesions in the inguinal regions, gluteal region, scrotum, and thighs, besides a superficial thrombophlebitis (Figure 1A–D). Due to the clinical severity of the disease, a high-frequency ultrasound (US) assessment using multifrequency high-resolution linear transducers (22 MHz) was performed, which revealed multiple intercommunicating fistulae and enlarged hair follicles in those regions. Altogether, these findings were scored as Hurley Score III (severe). The [^99m^Tc]Tc-anti-TNF-alpha whole-body imaging (Figure 1E,F) shows abnormal uptake in the gluteal region, scrotum, right thigh and in the venous tract in both lower limbs, in correlation with superficial thrombophlebitis described in the clinical exam. SPECT/CT images (Figure 1G) show abnormal uptake in the scrotum and the right intergluteal cleft.

Patient 2 (P2)

**Figure 2:**
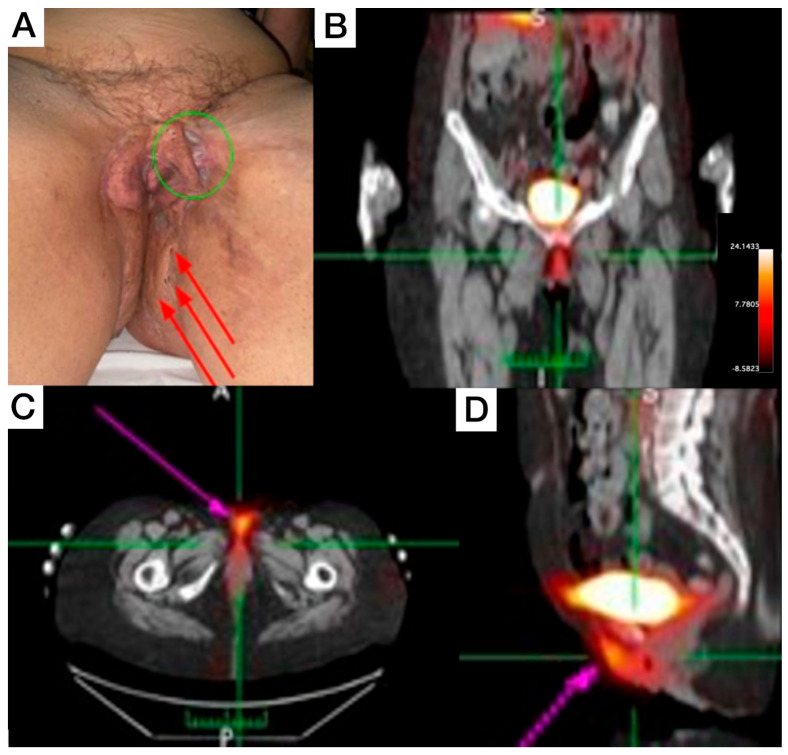
Patient 2 clinical (**A**) and SPECT/CT imaging findings (**B**–**D**). (**A**) Displays an extensive lesion affecting the vulva and perineum, presenting simultaneously in various evolutionary stages, including comedones, abscesses, and inactive cicatricial lesions (red arrows). On the anterolateral aspect of the left thigh root, an active lesion is visible, characterized by the presence of abscesses and a violaceous discoloration (green circle). This area also exhibits cicatricial tissue from a surgical anastomosis, which extends to the contralateral labium majus. (**B**–**D**) [^99m^Tc]Tc-anti-TNF-alpha scintigraphy SPECT/CT images show abnormal uptake in the genital and perineal regions (pink arrows).

Female, 66-year-old, diagnosis of HS for more than 50 years, with increasing severity over the last 10 years, using continuous antibiotic therapy (doxycycline 200 mg/day), and low-dose prednisone (5–10 mg/day). Clinical exam revealed evident end-stage scarring on external genitalia extending to inguinal regions and perineal areas, some comedones, and inactive fibrotic tissue. On the proximal anterolateral portion of the left thigh, a painful nodule (covered with erythematous to violaceous skin) was noticed. On the posterior view, inactive scarred tissue, macro-comedones, and interspersed erythema to healthy skin were detected on inter- and infra-gluteal regions. The patient underwent whole-body and SPECT [^99m^Tc]Tc-anti-TNF-alpha imaging, which revealed abnormal uptake in the genital area (Figure 2B), perineal (Figure 2C), and intergluteal region.

Patient 3 (P3)

**Figure 3:**
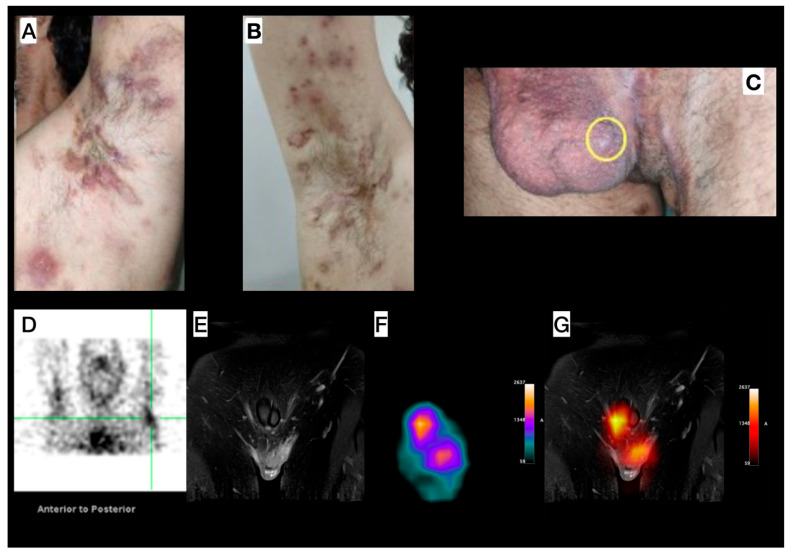
Patient 3—Left (**A**) and right (**B**) axillary region images in different disease stages, concomitantly (comedones, abscesses, inactive lesions, and scars). (**C**) Image of the genital and left inguinal region; scrotum presenting an inflammatory nodule (yellow circle). (**D**) SPECT image acquired from the head to the thoracic region, with the upper limbs elevated, showing abnormal uptake in both the right and left axillary (green reticle) regions. (**E**–**G**) Pelvic images: Magnetic Resonance Imaging (MRI) (**E**) shows focal scrotal wall thickening characterized by increased signal intensity on Short Tau Inversion Recovery (STIR) sequences and areas of enhanced contrast uptake, which are more prominent on the left side and extend into the inguinal regions. SPECT image (**F**) shows abnormal radiotracer uptake within the scrotum. (**G**) MRI and SPECT findings, enabling precise anatomical localization of the SPECT uptake.

Male, 33-year-old, diagnosed with HS for more than seven years, and with a diagnosis of acne for 17 years. Clinical exam revealed HS lesions in the abdomen, axillary regions (Figure 3A,B), inguinal regions, upper limbs, lower limbs, and gluteal region. A painful nodule was also found in the scrotum (Figure 3C). Doppler US and soft tissue MRI (axillary and inguinal regions) were performed, due to the severity of the case, for treatment orientation (adalimumab). MRI revealed focal scrotal wall thickening with increased signal intensity on Short Tau Inversion Recovery (STIR) sequences (Figure 3E) and areas of enhanced contrast uptake, more prominent on the left side, extending into the inguinal regions. Doppler US revealed small fluid collections in the scrotum and axillae, indicative of an active inflammatory process with fibrosis in both axillary regions. [^99m^Tc]Tc-anti-TNF-alpha imaging shows abnormal uptake in both axillary regions (Figure 3D) and scrotum (Figure 3E–G).

Patient 4 (P4)

**Figure 4:**
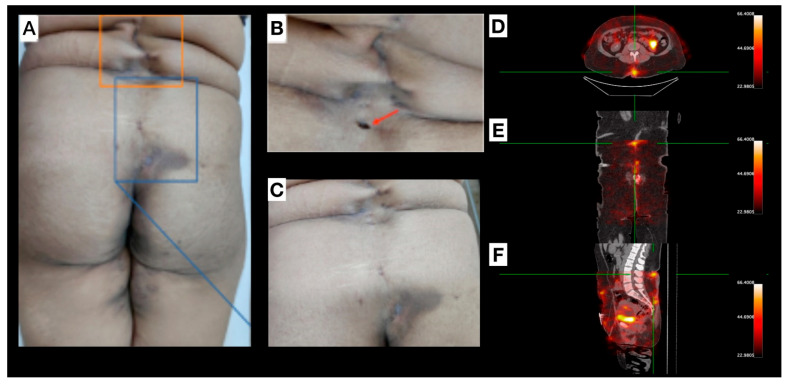
Patient 4 (P4): (**A**–**C**) depict lesions located within the lumbar and gluteal regions, originating from the L1 vertebral level and extending caudally to the sacral area. Interspersed within these affected areas, regions of apparently healthy tissue are observed, separating the discernible scars and clinically quiescent lesions. Image (**B**) highlights explicitly an oval-shaped opening (red arrow) representing a previous fistulous tract. A pilonidal cyst is delineated in the sacral region, with optimal visualization in image (**C**). (**D**–**F**): [^99m^Tc]Tc-anti-TNF-alpha SPECT/CT shows, in axial (**D**), coronal (**E**), and sagittal (**F**) views, abnormal uptake foci in the lumbar region, pilonidal cyst, and in a clinically inactive lesion (green reticle).

Female, 30 years old, diagnosed with HS for approximately one year. Clinical examination revealed several scar lesions on the inguinal region, vulva, gluteal region, and thighs (Figure 4). Mild inflammation signs were noticed on the lower-lumbar region (uncommon for HS manifestations), with sparse lesions from midline (L1-sacral region; Figure 4A–C). Non-inflammatory nodules, non-draining fistulae, post-inflammatory hyperchromic areas, and a previous fistulous tract oval-shaped ostium scar, with a depth deeper than the natural skin level, were also observed. [^99m^Tc]Tc-anti-TNF-alpha scintigraphy (Figure 4D–F) revealed abnormal uptake foci corresponding to both a pilonidal cyst and a clinically inactive lesion.

### 2.5. Scintigraphy Aspects

Consistent with clinical assessment, most of the active lesions exhibited abnormal radiotracer uptake during [^99m^Tc]Tc-anti-TNF-alpha scintigraphy, except for two lesions one in the inguinal region of patient 1 and the other on the left thigh of patient 2.

Abnormal uptake was identified in four regions by [^99m^Tc]Tc-anti-TNF-alpha scintigraphy that was not initially observed during clinical evaluation. In two of these regions, abnormal uptake was subsequently confirmed clinically in Patients 1 and 4. Of particular interest was the abnormal uptake identified within scar tissue in Patient 4, which was later confirmed as an active site upon re-evaluation. For the remaining two lesions, no clinical changes were noted, even after re-evaluation. Table 2 summarizes the scintigraphic findings in comparison to clinically identified lesions.

### 2.6. Statistical Analysis

Given the study’s limited sample size of four patients, the statistical analysis is primarily descriptive and qualitative, serving as a pilot, hypothesis-generating investigation rather than a definitive active inflammatory assessment, as no biopsy or histological examination was performed. The primary objective was to qualitatively describe abnormal [^99m^Tc]Tc-anti-TNF-alpha uptake in clinically inflammatory lesions, as this is considered in actual guidelines the main diagnosis assessment. Data collection encompassed patient demographics, detailed clinical findings, and qualitative assessments of scintigraphy uptake (including location of abnormal uptake and, when available, correlation with other imaging modalities). Statistical methods focused on presenting baseline characteristics using frequencies, percentages, means, and standard deviations, alongside detailed case-by-case narratives integrating clinical and imaging results to describe concordance and discordance. Crucially, no inferential statistical tests, such as p-values or confidence intervals, were performed due to the severe limitations in generalizability and statistical power; thus, specificity, sensitivity, and accuracy assessments are not feasible. This underscores that the findings are exploratory and not generalizable.

## 3. Discussion

Hidradenitis Suppurativa is a clinical condition that is part of the follicular occlusion tetrad, which can manifest in several parts of the body. A clinician makes the final diagnosis, and longitudinal follow-up is necessary for a more effective therapeutic approach [3]. During the physical examination, the disease can be evaluated as severe more easily than this could be classified through medical imaging or surgical approaches. Therefore, a better understanding and earlier disease activity profiling should result in a better outcome for the patient. Given that inflammatory processes are central to its pathogenesis, sensitive and specific detection methods are essential for identifying active lesions, particularly in severe cases where clinical evaluation may not fully capture disease activity.

In severe cases, the use of imaging modalities such as US and MRI scans is described, mainly for follow-up purposes; however, recent literature [22] shows its importance for accurate diagnosis of disease activity in this population, confirming the need for a sensitive and specific imaging method.

The US is the most common, feasible, low-cost, bedside/outpatient clinic assessment and does not require prior preparation for the exam. However, its efficacy is examiner- and site-dependent, exhibiting limited resolution for deep-seated lesions. This limitation can be exacerbated by the type of transducer employed, as high-definition transducers are not always readily available. Consequently, the US may demonstrate reduced accuracy in detecting subclinical and early-stage lesions [23].

Magnetic Resonance Imaging (MRI) offers excellent resolution for deep-seated lesions, making it particularly valuable for investigating fistulous tracts, especially in perianal disease [24]. However, MRI examinations are costly and provide limited resolution for superficial skin imaging [24]; therefore, they are not commonly indicated in all cases of HS. The confined space and prolonged stillness required during MRI acquisitions can also induce claustrophobia and discomfort.

Another consideration is the use of gadolinium-based contrast agents (GBCAs), administered in approximately 30–40% of MRI scans. While generally well-tolerated with rare anaphylactic reactions, GBCAs carry a potential risk of nephrotoxicity, particularly in patients with impaired renal function. Furthermore, recent studies have demonstrated gadolinium deposition in the brain and other tissues. Although no adverse health effects from this deposition have been definitively confirmed to date, its long-term implications remain under investigation [25]. In the present study, only Patient 3 underwent a pelvic MRI STIR-protocol with gadolinium administration after the [^99m^Tc]Tc-anti-TNF-alpha scintigraphy due to the severity of the disease and to obtain adalimumab (anti-TNF-alpha) free of charge through the Brazilian Public Health System (Sistema Único de Saúde—SUS), as clinical condition must be substantiated by imaging examination (MRI or ultrasonography) in these cases. MRI findings alterations were also observed in scintigraphic scans as abnormal uptake within the scrotum.

As HS can manifest diffusely and systemically, the ideal diagnostic imaging technique would offer simultaneous whole-body scanning for active lesions. Such a method should be examiner-independent and possess high sensitivity for minor tissue alterations to better assist clinicians in managing the disease. Scintigraphy imaging, as suggested by the preliminary results of this pilot study, could fulfill these crucial prerequisites.

The initial application of Nuclear Medicine in evaluating HS emerged as an incidental finding during 2-[^18^F]fluoro-2-deoxy-D-glucose ([^18^F]FDG) Positron Emission Tomography (PET) scans performed for cancer staging [26,27]. In these cases, subcutaneous sites demonstrating avid [^18^F]FDG uptake were identified not as metastatic patterns, but as manifestations of the patient’s HS lesions. To our knowledge, only three prior publications have utilized nuclear medicine techniques ([^18^F]FDG and [^67^Ga]Ga-citrate) for HS diagnosis and follow-up, both reporting successful results [26,27]. It is important to note that [^18^F]FDG and [^67^Ga]Ga-citrate are non-specific radiopharmaceuticals widely employed for imaging inflammation, and they are typically performed using different machines, technologies (often yielding higher imaging resolution), and protocols compared to conventional scintigraphy. Our objective in citing these examinations is to highlight that, despite potentially lower imaging resolution, [^99m^Tc]Tc-anti-TNF-alpha offers the significant advantage of targeting a specific molecule, given the crucial role of anti-TNF-alpha in the pathogenesis of the inflammatory process.

To our knowledge, this is the first imaging application of [^99m^Tc]Tc-anti-TNF-alpha in patients with HS, positioning this study as a proof-of-concept for a potential targeted molecular imaging strategy.

As previously mentioned, our research group has demonstrated that [^99m^Tc]Tc-anti-TNF-alpha is effective in detecting dermatological and rheumatological alterations [14,15,16,17,18,19]. Although the present study has a small cohort, we successfully employed our approach to demonstrate that this radiopharmaceutical may detect clinically active inflammatory sites. Despite the general concordance, as most clinically active lesions demonstrated abnormal radiotracer uptake during [^99m^Tc]Tc-anti-TNF-alpha scintigraphy, two clinically active lesions were not identified by [^99m^Tc]Tc-anti-TNF-alpha scintigraphy, i.e., the inguinal lesion in Patient 1 and the left thigh lesion in Patient 2. This discordance could be attributed to several factors. For instance, these lesions may represent a more fibrotic or chronic stage of inflammation, characterized by reduced active TNF-alpha expression, or limited radiopharmaceutical access due to tissue morphology. Conversely, two scintigraphic lesions (Patient 2, genital/perineal region, and Patient 4, pilonidal cyst/clinically inactive lesion) showed abnormal uptake that was not initially identified as clinically active upon reevaluation. This highlights the technique’s potential to detect previously missed clinically active lesions in a single examination. While two of these ‘unknown’ scintigraphic findings were later clinically confirmed, the nature of the persistent uptake in the others remains to be fully elucidated. This could potentially represent sub-clinical inflammation, early-stage disease activity that has not yet overtly manifested, or persistent molecular activity in areas of post-inflammatory remodeling. Wortsman et al. [22] demonstrated that clinical examination may not consistently reflect all pathological changes, particularly fluid collections, that are detectable with imaging modalities such as ultrasound. We assume that the scintigraphy could potentially provide similar diagnostic utility. However, for this hypothesis to be validated, a biopsy or histopathological examination of the lesion is necessary to confirm the findings observed on scintigraphy. Future studies with histological correlation are crucial to differentiate between active inflammation and radiopharmaceutical accumulation in areas of post-inflammatory change. Additionally, the scintigraphic findings were consistent with observations from available US and MRI examinations.

This study is subject to several important limitations. Firstly, the small cohort size (n = 4 patients) severely restricts the generalizability of our findings and precludes robust statistical analysis. Due to this limitation and the study’s pilot nature, we did not perform assessments of specificity, sensitivity, or accuracy, nor were we able to categorize scintigraphic findings by disease severity, or conduct quantitative explorations. The limited patient enrollment was primarily a consequence of conducting the study during the COVID-19 pandemic, which also hindered the expansion of our cohort.

Secondly, a comprehensive comparison between scintigraphy and other imaging modalities (US and MRI) across all identified lesions was not feasible, as not every lesion was assessed by each imaging technique. This prevents a direct, lesion-by-lesion comparative imaging analysis that would enhance the trustworthiness of our findings regarding diagnostic concordance.

A significant limitation of this pilot study is the absence of definitive histological confirmation, via biopsy, for all clinically identified lesions and scintigraphically abnormal uptake areas. While [^99m^Tc]Tc-anti-TNF-alpha scintigraphy has demonstrated potential for detecting inflammatory activity in other conditions, a robust correlation between tracer uptake and the presence of active cellular infiltrates in HS lesions necessitates histopathological assessment. This absence of biopsy further precluded definitive histological confirmation of the inflammatory status for all observed lesions, particularly for the two clinically active lesions not detected by scintigraphy and the areas of abnormal uptake within scar tissue. Although routine biopsy or histopathology are not standard practice in the clinical management of HS due to inherent challenges, such as patient discomfort and the diffuse nature of the disease, we recognize that incorporating biopsy will be a critical and necessary next step to evaluate the utility of this imaging method comprehensively. Future studies will, therefore, aim to include histological correlation where clinically feasible to establish this relationship more robustly.

Finally, the primary objective of this study was not to assess the efficacy of adalimumab in HS. Instead, we aimed to determine if [^99m^Tc]Tc-anti-TNF-alpha could detect active inflammatory sites, thereby serving as a proof-of-concept for its potential development as a theranostic agent to aid clinicians in identifying and guiding therapeutic strategies in HS patients in future research.

In summary, [^99m^Tc]Tc-anti-TNF-alpha scintigraphy represents a potential diagnostic tool for HS, particularly in severe cases with extensive lesion areas. Accurately evaluating all affected regions for active lesions poses a significant challenge for clinicians. This study demonstrates the technique’s capability to detect clinically active inflammatory sites specifically in patients with HS. Further studies are warranted with a larger cohort to establish potential quantitative cutoffs for abnormal uptake and to evaluate its future utility as a theranostic agent.

## 4. Materials and Methods

### 4.1. Patients

The present study was approved by the Institution’s Ethics Committee (protocol number: 07311013.0.0000.5257), and each participant signed the Informed Consent Form before [^99m^Tc]Tc-anti-TNF-alpha scintigraphy.

Patients were assessed from January 2019 until December 2022 using the Hurley Scoring system and Hidradenitis Suppurativa Physician Global Assessment (HS-PGA). Subsequently, patients underwent photographic documentation and then [^99m^Tc]Tc-anti-TNF-alpha scintigraphy.

The inclusion criteria were subjects who were already under regular clinical follow-up at the Dermatology Outpatient Unit, not currently treated with any immunosuppressants (methotrexate ≥ 20 mg/week; prednisone ≥ 20 mg/day) and/or immunobiological agents. All other therapies used for symptom control, such as analgesics and non-hormonal anti-inflammatories, were accepted.

Subjects with autoimmune diseases, ischemic heart disease, pregnancy, active tuberculosis, and/or inflammatory skin disorder other than HS were excluded.

To further ensure patient safety, hospitalization beds were reserved for immediate care in case any enrolled patients experienced adverse effects. Additionally, patients were provided with the clinician’s contact information to report any post-procedure side effects or concerns. It is worth noting that no adverse effects have been observed to date in patients who underwent [^99m^Tc]Tc-anti-TNF-alpha scintigraphy for other indications in prior studies. Consequently, we do not anticipate significant side effects or hazard risks in the current study.

### 4.2. [^99m^Tc]Tc-Anti-TNF-Alpha Scintigraphy

For [^99m^Tc]Tc-anti-TNF-alpha scintigraphy, monoclonal human anti-TNF-alpha (150 µL) (Adalimumab, Humira^®^; Abbott Laboratories, North Chicago, IL, USA) was labelled with [^99m^Tc]pertechnetate, using the same protocol as used in other previously studies described by our group [14,15,16,17,18,19]. Briefly, anti-TNF-alpha was incubated for 10 min with a reducing agent (stannous chloride), and then 370 MBq (10 mCi) of [^99m^Tc]pertechnetate was added. Thereafter, the labelled antibody was sterilized by filtering the solution through a 0.22-µm Millipore sterile filter (Merck KGaA—Millipore^®^, Darmstadt, Germany). Before administration, and in accordance with institutional protocol, quality control was performed to confirm the radiopharmaceutical’s labeling efficiency and radiochemical purity. This included ascending instant thin-layer chromatography (ITLC) on Whatman No. 4 paper (using acetone and 0.9% NaCl as mobile phases for free [^99m^Tc]pertechnetate and hydrolyzed-reduced [^99m^Tc] assessment, respectively). Administration was permitted only when a labeling efficiency of 90% or higher was achieved. The total amount of anti-TNF-alpha administered was 15% of a treatment dose, which is insufficient to elicit a pharmacological effect. [^99m^Tc]Tc-anti-TNF-alpha scintigraphy images were obtained 30 min after intravenous injection of 370 MBq (10 mCi) [^99m^Tc]Tc-anti-TNF-alpha. Whole-body and single-photon emission computed tomography (SPECT) slices were performed for the regions of interest. The scintigraphy was considered positive when an abnormal uptake was observed outside physiological biodistribution (urinary system).

Whole-body images were acquired using a Millennium MG gamma camera (GE Medical Systems, Milwaukee, WI, USA) equipped with a high-resolution, low-energy collimator. The acquisition parameters included a 15% energy window centered at 140 KeV and a 1024 × 256 matrix. Patients were initially positioned as supine, with the camera detector oriented for both anterior and posterior views. A second image acquisition was performed with patients in a standing position, with feet positioned wider than hip width. To mitigate potential contamination and subsequent false-positive results, particularly given the common involvement of inguinal and genital regions in HS, all subjects were instructed to void before radiopharmaceutical injection and before proceeding with the imaging protocols. SPECT images were acquired using a 256 × 256 matrix.

Acquired images were qualitatively evaluated for abnormal uptake occurring outside the expected physiological biodistribution. Uptake was considered normal if it corresponded to known physiological biodistribution patterns, and abnormal if it was observed in unexpected sites. All images were qualitatively analyzed by two board-certified nuclear medicine physicians. These physicians were aware of the patients’ underlying Hidradenitis Suppurativa (HS) condition but were blinded to the specific location and severity of individual lesions.

After low-dose SPECT acquisition, patients underwent CT scanning for anatomical reference, using the PET/CT OPTIMA 560 (GE Medical Systems, Milwaukee, WI, USA) at the University Nuclear Medicine Department. Acquired images were merged with the Osirix MD software (version 12.0.4).

## 5. Conclusions

In conclusion, [^99m^Tc]Tc-anti-TNF-alpha scintigraphy demonstrated the ability to detect increased radiotracer uptake in most clinically identified active lesions in this pilot cohort. Furthermore, it identified additional foci of potential inflammatory activity in various anatomical regions, including sites not initially considered clinically active. This study represents the first reported application of [^99m^Tc]Tc-anti-TNF-alpha for imaging diagnostic evaluation of HS, positioning it as a proof-of-concept for a targeted molecular imaging strategy in this complex disease. While promising, the inherent limitations of this pilot study, particularly the small sample size and absence of histological correlation, underscore the need for larger, prospective studies to definitively establish its utility as a diagnostic and theranostic tool in detecting clinically active inflammatory sites in HS patients.

## 6. Patents

The [^99m^Tc]Tc–anti-TNF-alpha labelling technique used in this study is patented under registration at INPI, Brazil. Gutfilen, B.; Lopes FPPL; et al. Patent: Privilégio de Inovação. Processo indireto de marcação de anticorpos monoclonais ou policlonais associados a doenças imunológicas com radionuclídeos; reagente para diagnóstico ‘in vivo’ ou ‘in vitro’ a base de anticorpos monoclonais ou policlonais marcados com radionuclídeos; uso do reagente para diagnóstico, método de diagnóstico ‘in vitro’ e ‘in vivo’ de processos imunológicos e kit para diagnóstico de processos imunoló. Patent Number: PI02056810, Registering authority: INPI—Instituto Nacional da Propriedade Industrial, Application Granted Date: 4 October 2002.

## Figures and Tables

**Table 1:** Baseline characteristics of the patients.

Characteristics	HS Patients (n = 4)
Age (years), mean (SD)	41 (±7.14)
Male, n (%)	2 (50%)
Race, white (%)	3 (75%)
Smoking habit, n (%), yes	1 (25%)
Overweight, n (%), yes	3 (75%)
Hurley stage	
II (moderate), n (%)	2 (50%)
III (severe), n (%)	2 (50%)
HS-PGA	
5 (Very severe)	1 (25%)
4 (Severe)	2 (50%)
1 (Minimal)	1 (25%)

**Table 2:** Comparison of scintigraphic findings and clinically identified lesions.

Patient ID	Localization	Clinically Identified Lesions	[^99m^Tc]Tc-Anti-TNF-Alpha Scintigraphy Findings (Abnormal Uptake)
1	Inguinal regions	Yes, active lesion	No
	Gluteal region	Yes, active lesion	Yes
	Scrotum	Yes, active lesion	Yes
	Right Thigh	Yes, active lesion	Yes
	Lower limbs	Yes, superficial thrombophlebitis (subsequently identified upon re-evaluation of the patient, a reassessment initiated due to findings from the scintigraphy).	Yes
2	Genital region	No active lesions	Yes
	Perineal region	No active lesions	Yes
	Inguinal region	No active lesions	No
	Left thigh	Yes, active lesion	No
	Gluteal region	Yes, active lesions	Yes
3	Thoracic region	No active lesions	No
	Axillary regions	Yes, active lesion	Yes
	Upper limbs	No active lesions	No
	Abdomen region	No active lesions	No
	Inguinal region	Yes, active lesion	Yes
	Scrotum	Yes, active lesion	Yes
	Lower limbs	No active lesions	No
	Gluteal region	No active lesions	No
4	Inguinal region	No active lesion	No
	Vulva	No active lesion	No
	Thighs	No active lesion	No
	Gluteal region	No active lesion	No
	Lower lumbar region	Yes, active lesion (subsequently identified upon re-evaluation of the patient, a reassessment initiated due to findings from the scintigraphy)	Yes (pilonidal cyst and a clinically inactive lesion)

## Data Availability

All data generated or analysed during this study are included in this article. Further inquiries can be directed to the corresponding author.

## Abbreviations

The following abbreviations are used in this manuscript:

HS	Hidradenitis Suppurativa
HS-PGA	Hidradenitis Suppurativa-Physician’s Global Assessment (HS-PGA)
MRI	Magnetic Resonance Imaging
CT	Computed Tomography
SPECT	Single Photon Emission Computed Tomography
US	Ultrasound
TNF	Tumor Necrosis Factor
FDG	fluoro-2-deoxy-D-glucose
GBCA	gadolinium-based contrast agents
